# The complete chloroplast genome of *Saposhnikovia divaricata*

**DOI:** 10.1080/23802359.2019.1704200

**Published:** 2019-12-27

**Authors:** Zhenzhen Bao, Ziyan Zhu, Haijiang Zhang, Yuan Zhong, Weiqi Wang, Jingzheng Zhang, Jie Wu

**Affiliations:** a School of pharmacy, Jiangsu Health Vocational College, Nanjing, P. R. China;; b School of Traditional Chinese Pharmacy, China Pharmaceutical University, Nanjing, China;; c Jiangsu Key Laboratory of Regional Resource Exploitation and Medicinal Research, Huaiyin Institute of Technology, Huaian, PR China

**Keywords:** *Saposhnikovia divaricata*, complete chloroplast genome, phylogenetic analysis

## Abstract

*Saposhnikovia divaricata* (Trucz.) Schischk. is a traditional Chinese herbal medicine widely distributed in Eastern Siberia and Northern Asia. In this research, we assembled and characterized the complete chloroplast genome sequence of *S. divaricata* from high-throughput sequencing data. The chloroplast genome was 147,834 bp in length, consisting of large single-copy (LSC) and small single-copy (SSC) regions of 93,202 bp and 17,324 bp, respectively, which were separated by a pair of 18,654 bp inverted repeat (IR) regions. The genome is expected to contain 129 genes, including 85 protein-coding genes, 36 tRNA genes, and 8 rRNA genes. The total GC content of the genome is 37.5%. A phylogenetic tree reconstructed by 40 chloroplast genomes reveals that *S. divaricata* is mostly related to *Ledebouriella seseloides*.

*S. divaricata*, the sole species of the genus Saposhnikovia Schischk, is widely distributed in Eastern Siberia and Northern Asia (Pei et al. 2002). The roots of *S. divaricata*, named “Fangfeng” in Chinese, is a traditional Chinese herbal medicine commonly used in the treatment of arthralgia, rheumatism (Kreiner et al. 2017), stroke, headaches, fever, colds, etc (Qian et al. 2010). Pharmacological analysis indicated that *S. divaricata* has the ability to anti-convulsant, analgesic (Okuyama et al. 2001), anti-cancer, anticoagulant, anti-inflammatory (Yu et al. 2015), and antipyretic activities (Guo et al. 2001) etc. However, its phylogenetic position is still unclear due to the lack of genomic resources of genus Saposhnikovia Schischk in previous researches. In this study, we determined the complete chloroplast genome of *S. divaricata* by using high throughput sequencing technology, which will provide useful information for the phylogeny of genus Saposhnikovia Schischk and the further research on evolution of Saposhnikovia.

The total genomic DNA was extracted from the fresh leaves of *S. divaricata* (32°076' N, 118°612' E) using the DNeasy Plant Mini Kit (Qiagen, Valencia, CA, USA). The species were stored in Jiangsu Health Vocational College with the accession number of FF20190911BZZ-17. The DNA was stored at −80 °C in our lab. The whole genome sequencing was conducted by Nanjing Genepioneer Biotechnologies Inc. (Nanjing, China) on the Illumina Hiseq platform. The filtered sequences were assembled using the program SPAdes assembler 3.10.0 (Bankevich et al. 2012). Annotation was performed using the DOGMA and BLAST searches (Wyman et al. 2004).

The cp genome of *S. divaricata* was determined to comprise a 147,834 bp double stranded, circular DNA (GenBank accession no. MN539269), which containing two inverted repeat (IR) regions of 18,654 bp, separated by large single-copy (LSC) and small single-copy (SSC) regions of 93,202 bp and 17,324 bp, respectively. The overall GC content of *S. divaricata* cp genome is 37.5% and the corresponding values in LSC, SSC and IR regions are 35.9%, 30.8% and 44.6%, respectively. The cp genome was predicted to contain 129 genes, including 85 protein-coding genes, 36 tRNA genes, and 8 rRNA genes. Three protein-coding genes, six tRNA genes and four rRNA genes were duplicated in IR regions. Eighteen genes contained two exons and four genes (clpP, ycf3 and two rps12) contained thee exons.

To investigate its taxonomic status, alignment was performed on the 40 chloroplast genome sequences using MAFFT v7.307 (Katoh and Standley 2013), and a maximum likelihood (ML) tree was constructed by FastTree version 2.1.10 (Price et al. 2010). As expected, *S. divaricata* is mostly related to *L. seseloides*, with bootstrap support values of 100% (Figure 1). The complete cp genome sequence of *S. divaricata* will provide a useful resource for the conservation genetics of this species as well as for the phylogenetic studies of Saposhnikovia Schischk genus.

**Figure 1.**
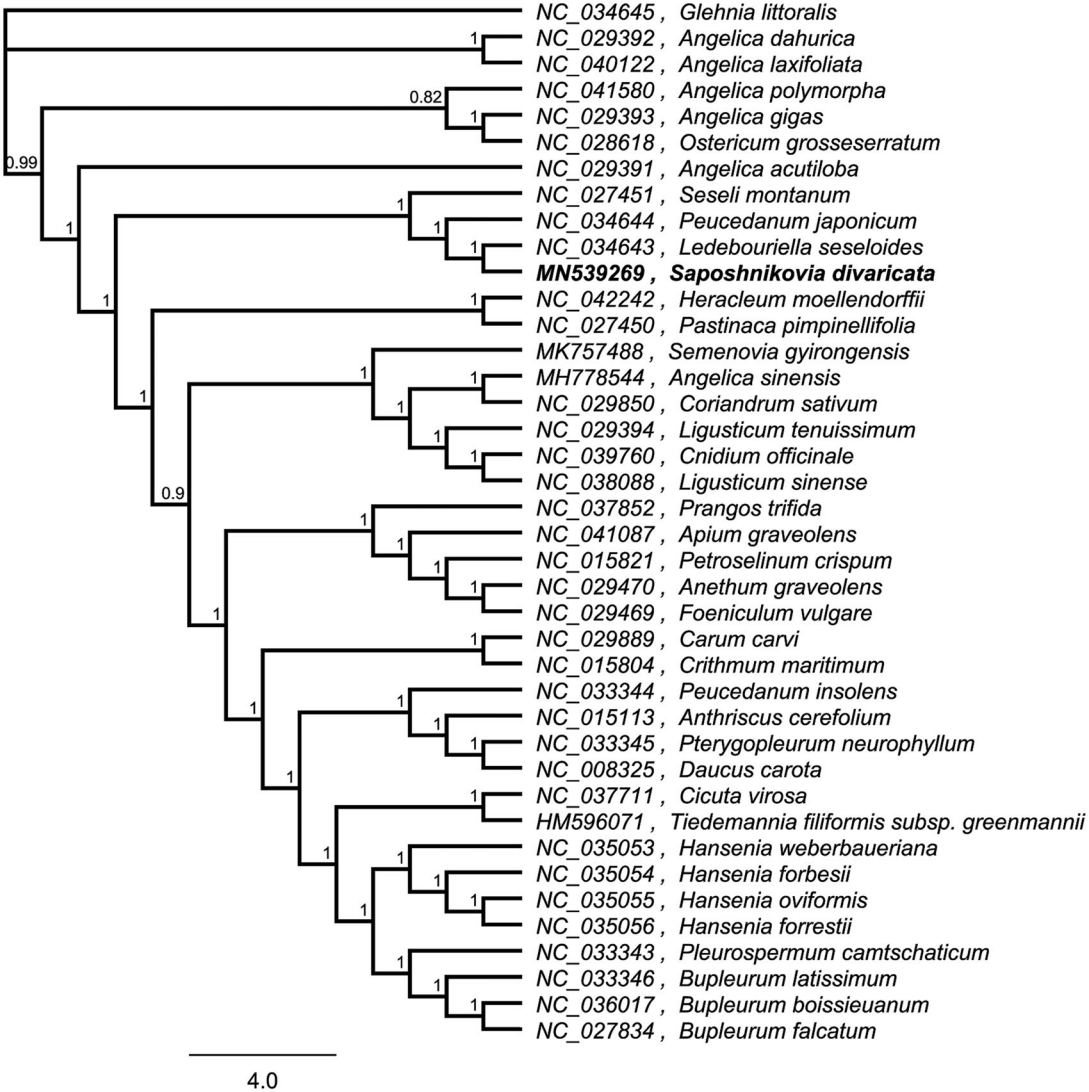
Phylogenetic tree inferred by Maximum Likelihood (ML) method based on 40 representative species. A total of 1000 bootstrap replicates were computed and the bootstrap support values are shown at the branches. GenBank accession numbers were shown in Figure 1.
